# Assessment of Sensory Impairment and Health Care Satisfaction Among Medicare Beneficiaries

**DOI:** 10.1001/jamanetworkopen.2020.25522

**Published:** 2020-11-13

**Authors:** Lama Assi, Ahmed F. Shakarchi, Orla C. Sheehan, Jennifer A. Deal, Bonnielin K. Swenor, Nicholas S. Reed

**Affiliations:** 1 Wilmer Eye Institute, Johns Hopkins University School of Medicine, Baltimore, Maryland; 2 Cochlear Center for Hearing and Public Health, Johns Hopkins Bloomberg School of Public Health, Baltimore, Maryland; 3 Johns Hopkins Center on Aging and Health, Baltimore, Maryland; 4 Division of Geriatric Medicine and Gerontology, Johns Hopkins University School of Medicine, Baltimore, Maryland; 5 Department of Epidemiology, Johns Hopkins Bloomberg School of Public Health, Baltimore, Maryland

## Abstract

**Importance:**

Satisfaction with care is associated with improved quality of care and health outcomes. Sensory impairment can be a barrier to effective communication and access to care, and this may result in reduced satisfaction with care.

**Objective:**

This study examined the association between sensory impairment and health care satisfaction among Medicare beneficiaries.

**Design, Setting, and Participants:**

This cross-sectional study used data from the 2017 Medicare Current Beneficiary Survey (MCBS), a nationally representative in-person survey of Medicare beneficiaries. Functional sensory impairment was categorized as no sensory impairment, hearing impairment, vision impairment, and dual sensory impairment. Patient dissatisfaction included responses on quality of care, ease to get to a doctor, out-of-pocket costs paid, information given, and doctors’ concern with overall health rather than an isolated symptom or disease.

**Exposures:**

Self-reported functional sensory impairment.

**Main Outcomes and Measures:**

Multivariable-adjusted odds ratios (ORs) of the association between dissatisfaction with care and sensory impairment.

**Results:**

A total of 10 783 respondents representing 44 736 889 Medicare beneficiaries (8944 [85.3%] aged ≥65 years, 5733 [52.9%] women, and 8195 [75.5%] non-Hispanic White) were included. Dual sensory impairment compared with no sensory impairment was associated with the highest odds of dissatisfaction across outcomes, including quality of care (OR, 1.52; 95% CI, 1.12-2.08). Compared with no sensory impairment, having dual sensory impairment (OR, 1.82; 95% CI, 1.40-2.37), hearing impairment (OR, 1.67; 95% CI, 1.29-2.17), or vision impairment (OR, 1.56; 95% CI, 1.18-2.08) were associated with dissatisfaction with the information provided about what was wrong. Those with hearing impairment (OR, 1.38; 95% CI, 1.03-1.86) or dual sensory impairment (OR, 2.03; 95% CI, 1.55-2.66) were more likely to be dissatisfied with doctors’ concern with overall health compared with those with no sensory impairment. Having dual sensory impairment or vision impairment only was associated with greater odds of dissatisfaction with ease to get to a doctor (dual sensory: OR, 1.69; 95% CI, 1.24-2.30; vision: OR, 1.63; 95% CI, 1.14-2.31) and out-of-pocket costs paid (dual sensory: OR, 1.27; 95% CI, 1.04-1.54; vision: OR, 1.31; 95% CI, 1.07-1.61).

**Conclusions and Relevance:**

These findings contribute to the growing body of literature on sensory impairment and patient satisfaction and have implications for health care system planning and spending to provide patient-centered care for older adults.

## Key Points

**Question:**

What is the association between hearing, vision, or dual sensory impairment and dissatisfaction with health care among Medicare beneficiaries?

**Findings:**

In this cross-sectional study including 10 783 Medicare beneficiaries, people with dual sensory impairment were more likely to report dissatisfaction with quality of care, communication from health care professionals, and access to care, and those with hearing and vision impairment were more likely to report dissatisfaction with the information given by health care professionals than those without sensory impairments.

**Meaning:**

Addressing sensory impairment across health care settings via quality initiatives and environmental modifications may represent an area of intervention to improve health care satisfaction.

## Teaser

This cross-sectional study assesses the association between hearing, vision, or dual sensory impairment and patient satisfaction with quality of care, communication from health professionals, and access to care among a nationally representative sample of Medicare beneficiaries.

## Introduction

The Picker Institute has identified 8 dimensions of patient-centered care, which include respect for the patient’s values and needs, information and education, and access to care.^1^ Patient-centered care is vital to patient satisfaction with care, an increasingly important metric in health care that is associated with several quality-of-care measures, including better surgical outcomes^2^ and reduced health care spending,^3^ readmission rates, and mortality.^2,4^ In recent years, the Centers for Medicare & Medicaid Services emphasized patient satisfaction with care by linking it to the hospital value-based purchasing program.^5^

Sensory impairment may be a barrier to effective patient-centered care, affecting health care professionals’ communication with patients and access to care.^6,7,8^ In the US, about 9% of adults older than 70 years have noncorrectable vision impairment,^9^ and up to two-thirds have bilateral hearing impairment.^10^ The prevalence of both impairments increases with age, and concurrent vision and hearing impairment (ie, dual sensory impairment) is estimated to affect 1 in 9 adults over the age of 80 years.^11^ Recent epidemiologic literature has associated sensory loss with important gerontological outcomes that include functional and cognitive decline,^12,13,14,15,16,17^ worse quality of life,^18,19,20^ and increased mortality.^21,22,23^ Moreover, sensory impairment has been associated with higher health care utilization and costs^24,25,26,27^ and with difficulty communicating with health care professionals and accessing health information.^7,8,28,29,30,31^ People with vision impairment have also reported physical access barriers, including getting to and around physicians’ offices.^31^

The combined effects of access to care barriers, poor communication with health care professionals, and higher health resource needs and costs may contribute to reduced patient satisfaction with care among adults with sensory impairment. Previous work has focused on the association of individual sensory impairments and satisfaction with care, particularly with hearing impairment among older adults.^28,32^ Few articles have focused on vision impairment specifically^30,33^ and none have addressed dual sensory impairment in terms of general health care satisfaction. Dual sensory impairment may exacerbate the impact of either individual sensory impairment, as it strains an individual’s ability to use sensory compensation strategies.

Given the paucity of research on vision and dual sensory impairment, we explored the association of functional sensory impairment and self-reported satisfaction with care among Medicare beneficiaries using the 2017 Medicare Current Beneficiary Survey (MCBS).^34^

## Methods

### Study Population

The MCBS is an ongoing nationally representative survey of Medicare beneficiaries used to monitor and evaluate Medicare, the US health insurance program for adults 65 years and older, and younger persons with a qualifying disability. The MCBS has a rotating panel design, and each panel of sampled beneficiaries is interviewed in person 3 times per year for 4 consecutive years. Analyses for this study used data from the 2017 Fall round among 11 771 community-dwelling adults who were not diagnosed with dementia and who used health care services in the past year. The institutional review board of NORC at the University of Chicago approved the protocol and consent procedures of the MCBS. Reporting of this study followed the Strengthening the Reporting of Observational Studies in Epidemiology (STROBE) reporting guideline for cross-sectional studies.^35^

### Outcomes Measures

The primary outcome was patient dissatisfaction with care. Participants were asked about their satisfaction with the quality of care received (ie, “Please tell me how satisfied or dissatisfied you have been with the overall quality of the health care you have received over the past year”). Dissatisfaction with care was categorized as a binary outcome: satisfied (“satisfied” or “very satisfied”) or dissatisfied (“dissatisfied” or “very dissatisfied”). Secondary outcomes included dissatisfaction with 4 aspects of care: ease to get to a doctor, out-of-pocket costs paid, information given about what was wrong, and doctors’ concern with overall health rather than an isolated symptom or disease.

### Sensory Impairment

The primary exposure was functional sensory impairment. Participants were asked to describe their vision and hearing by selecting 1 of the statements: “no trouble,” “a little trouble,” or “a lot of trouble” seeing and/or hearing. Functional vision impairment was defined as any self-reported trouble seeing (“a little trouble” or “a lot of trouble”) with the use of glasses or contact lenses when applicable. Functional hearing impairment was defined as any self-reported trouble hearing with the use of hearing aids when applicable. Sensory impairment was categorized as: no sensory impairment, hearing impairment only, vision impairment only, and dual sensory impairment.

### Other Measures

Covariates that may confound the association between sensory impairment and satisfaction with care were identified in the satisfaction with care and disability literature.^28,30,33^ Sociodemographic and access-to-care characteristics included age (<65 years, 65-75 years, >75 years), sex, race and ethnicity (non-Hispanic White, non-Hispanic Black, Hispanic, other), educational attainment (less than high school, high school or equivalent, more than high school), marital status, metropolitan area status, income poverty ratio Medicare threshold (≤100% of the federal poverty level [FPL], >100% and ≤120%, >120% and ≤135%, >135% and ≤200%, and >200%), and supplemental insurance coverage (including Medigap, employer-sponsored plans, and plans directly purchased).

General health determinants included a comorbidity count (0, 1-2, 3-5, and ≥6 conditions), derived as a count of self-reported physician diagnoses of chronic conditions (including heart disease, myocardial infarction, hypertension, diabetes, stroke, arthritis, cancer, chronic lung disease, mental disorder, and depression), and number of limitations in activities of daily living (ADLs) and instrumental activities of daily living (IADLs) because of health issues (categorized as none, IADLs only, 1-2 ADLs, 3-4 ADLs, and 5-6 ADLs).

### Statistical Analysis

Respondents with missing information about the exposure (125 respondents) or covariates (863 respondents) were excluded. Analyses accounted for the complex survey design of MCBS and survey nonresponse, using the recommended replicate weight approach to variance estimation and weights provided by CMS.^34^ Population characteristics were described by sensory impairment status. Multivariable logistic regression models were used to explore the association between sensory impairment and dissatisfaction outcomes. Given Medicare’s inclusion of working-age adults with a qualifying disability, models were repeated after excluding those younger than 65 years old to ensure that they did not significantly change the results. Coefficients were converted into odds ratios (ORs) and reported with a 95% confidence interval. The *P* values were 2-tailed with statistical significance set at *P* < .05. All analyses were conducted using R, version 3.6.1 (R Project for Statistical Computing).

## Results

### Population Characteristics

A total of 10 783 community-dwelling respondents, representing a weighted sample of 44 736 889 Medicare beneficiaries, were included. Of these respondents, 8944 (85.3%) were aged 65 years and older, 5733 (52.9%) were female, and 8195 (75.5%) were non-Hispanic White. Overall, 4250 (40.9%) reported no sensory impairment, 2822 (25.4%) hearing impairment only, 1557 (14.8%) vision impairment only, and 2154 (18.9%) dual sensory impairment (Table 1). A greater proportion of persons with dual sensory impairment than without lived in nonmetropolitan areas and had chronic conditions and functional limitations. Those with vision impairment had the greatest proportion of people younger than 65 years and having an income poverty ratio 100% or below the FPL, while those with hearing impairment had the greatest proportion of adults over the age of 75 years, and having an income poverty ratio greater than 200% FPL.

**Table 1.** Population Characteristics by Sensory Impairment Status

Characteristic	Responses, No. (weighted %)					*P* value^b^
	Overall^a^	Sensory impairment				
		None	HI Only	VI Only	DSI	
Total	10 783 (100)	4250 (40.9)	2822 (25.4)	1557 (14.8)	2154 (18.9)	NA
Age, y						
<65	1839 (14.7)	767 (13.2)	2822 (8.2)	1557 (27.3)	2154 (17.1)	<.001
65-74	3687 (52.3)	1628 (57.6)	246 (51.5)	486 (48.0)	340 (45.3)	
≥75	5257 (33.0)	1855 (29.2)	909 (40.3	506 (24.7)	644 (37.7)	
Women	5733 (52.9)	2390 (56.2)	1278 (44.4)	940 (59.7)	1125 (51.7)	<.001
Race/ethnicity						
Non-Hispanic						<.001
White	8195 (75.5)	3132 (73.4)	2368 (83.7)	1029 (67.4)	1666 (75.3)	
Black	1075 (9.6)	489 (10.8)	144 (5.0)	250 (14.6)	192 (9.0)	
Hispanic	869 (7.9)	396 (9.3	160 (5.1)	171 (10.0)	142 (7.1)	
Non-Hispanic other^c^	644 (7.0)	233 (6.5)	150 (6.2)	107 (8.0)	154 (8.5)	
Education						
<High school	1803 (14.3)	664 (13.0)	375 (10.6)	353 (20.7)	411 (17.0)	<.001
High school or equivalent	3837 (32.9)	1445 (30.5)	1021 (33.9)	568 (34.1)	803 (35.9)	
>High school	5143 (52.8)	2141 (56.5)	1426 (55.5)	636 (45.3)	940 (47.1)	
Not married	5586 (45.5)	2196 (45.0)	1308 (39.2)	895 (52.2)	1187 (49.9)	<.001
Nonmetropolitan area	2702 (19.9)	987 (17.7)	709 (20.6)	379 (18.8)	627 (24.5)	<.001
Income poverty ratio Medicare threshold, % of FPL						
≤100	1904 (14.5)	795 (14.5)	323 (9.5)	390 (20.1)	396 (16.6)	<.001
>100 to ≤120	665 (5.5)	263 (5.2)	134 (4.1)	124 (7.6)	144 (6.5)	
>120 to ≤135	528 (4.3)	201 (4.2)	126 (3.8)	85 (4.6)	116 (5.2)	
>135 to ≤200	1749 (15.6)	628 (13.9)	462 (15.3)	254 (16.9)	405 (18.5)	
>200	5937 (60.1)	2363 (62.2)	1777 (67.4)	704 (50.8)	1093 (53.3)	
No supplemental insurance coverage	5508 (46.9)	2188 (45.6)	1251 (39.9)	945 (56.4)	1124 (51.5)	<.001
Chronic conditions, No.^d^						
0	740 (7.5)	421 (10.5)	164 (6.9)	81 (5.2)	74 (3.4)	<.001
1-2	4208 (41.0)	1958 (48.5)	1065 (39.4)	555 (38.2)	630 (29.0)	
3-5	5176 (45.4)	1722 (37.8)	1442 (48.3)	799 (48.5)	1213 (55.8)	
>5	659 (6.2)	149 (3.3)	151 (5.5)	122 (8.1)	237 (11.8)	
Functional limitations						
None	5929 (59.8)	2773 (70.7)	1683 (65.2)	677 (47.5)	796 (38.7)	<.001
IADLs						
Only	1717 (13.8)	611 (11.7)	430 (13.1)	281 (16.0)	395 (17.4)	
1-2	2123 (17.9)	646 (13.1)	512 (15.6)	375 (22.1)	590 (28.1)	
3-4	660 (5.6)	142 (2.8)	141 (4.5)	136 (9.2)	241 (10.2)	
5-6	354 (2.9)	78 (1.7)	56 (1.6)	88 (5.2)	132 (5.7)	

Abbreviations: DSI, dual sensory impairment; FPL, Federal Poverty Level; HI, hearing impairment; IADL, instrumental activities of daily living; VI, vision impairment.

^a^ Representing 44 736 889 Medicare beneficiaries.

^b^ Groupwise comparisons using χ^2^ tests of association for survey data.

^c^ Racial/ethnic groups included in other were American Indian or Alaska Native, Asian, and Native Hawaiian or other Pacific Islander.

^d^ Number of chronic conditions derived as a count of self-reported physician diagnoses of the following conditions: heart disease, myocardial infarction, hypertension, diabetes, stroke, arthritis, cancer, chronic lung disease, mental disorder, and depression.

### Dissatisfaction With Quality of Care

Overall, the majority of Medicare beneficiaries were either very satisfied or satisfied with the quality of care received (Figure). The highest percentage of dissatisfaction with quality of care was among those with dual sensory impairment (139 of 2154 respondents; weighted percentage, 6.7%), followed by vision impairment only (80 of 1557 respondents; weighted percentage, 5.3%), hearing impairment only (106 of 2822 respondents; weighted percentage, 3.8%), and no sensory impairment (134 of 4250 respondents; weighted percentage, 3.1%).

**Figure.**
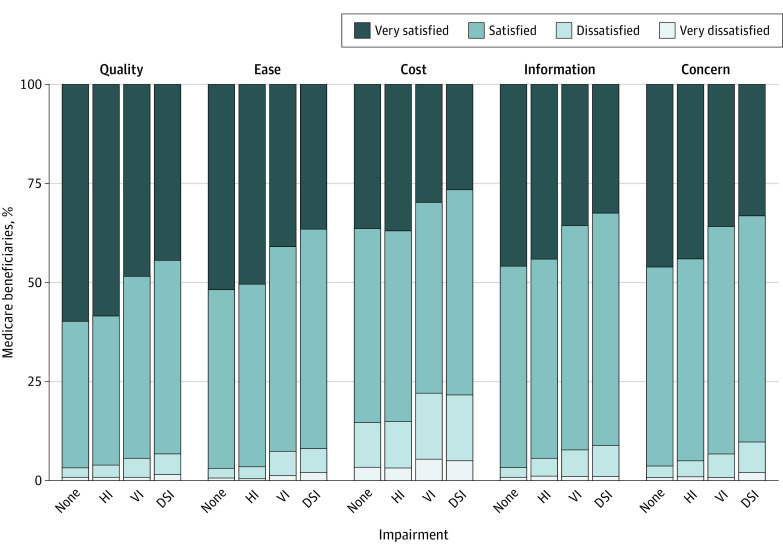
Satisfaction With Overall Quality and Different Aspects of Care by Sensory Impairment Status in the 2017 Medicare Current Beneficiary Survey Responses totaled 10 783, representing 44 736 889 Medicare beneficiaries. Participants were asked about their satisfaction with the overall quality of the health care received in the past year (quality), the ease and convenience of getting to a doctor or other health professional from where they live (ease), the out-of-pocket costs they paid for health care (cost), the information they were given about what was wrong with them (information), and the concern of doctors or other health professionals for their overall health rather than just for an isolated symptom or disease (concern). DSI indicates dual sensory impairment; HI, hearing impairment; and VI, vision impairment.

In the adjusted model, those with dual sensory impairment had higher odds of dissatisfaction with quality of care relative to no sensory impairment (OR, 1.52; 95% CI, 1.10-2.10; *P* = .01); the odds were were not significantly higher for those with hearing impairment (OR, 1.31; 95% CI, 0.88-1.95; *P* = .19) and vision impairment only (OR, 1.17; 95% CI, 0.79-1.74; *P* = .43) (Table 2). In the same model, those who were unmarried (vs married: OR, 1.43; 95% CI, 1.06-1.92; *P* = .02) were more likely to be dissatisfied with the quality of care received. The difference between those with any number of functional limitations (eg, IADLs only) vs no limitations was not statistically significant (OR, 1.44; 95% CI, 0.97-2.12; *P* = .07). Being 65 years or older was associated with lower odds of dissatisfaction with quality of care (age >75 years vs <65 years: OR, 0.52; 95% CI, 0.37-0.72; *P* < .001); an income poverty ratio between 120% and 135% or equal to or greater than 200% (vs ≤100% of the FPL: OR, 1.12; 95% CI, 0.73-1.72; *P* = .06) was not associated with lower odds of dissatisfaction with quality of care.

**Table 2.** Multivariable-Adjusted Odds Ratios of Dissatisfaction With Overall Quality of Care Over the Previous Year in the 2017 Medicare Current Beneficiary Survey

Characteristic	OR (95% CI)	*P* value
Sensory impairment		
No sensory impairment	1 [Reference]	>.99
Hearing only	1.31 (0.88-1.95)	.19
Vision only	1.17 (0.79-1.74)	.43
Dual sensory	1.52 (1.10-2.10)	.01
Age, y		
<65	1 [Reference]	>.99
65 to 74	0.68 (0.48-0.95)	.03
>74	0.52 (0.37-0.72)	<.001
Women	1.18 (0.94-1.47)	.16
Race/ethnicity		
Non-Hispanic White	1 [Reference]	>.99
Non-Hispanic Black	1.13 (0.77-1.65)	.53
Hispanic	1.20 (0.74-1.94)	.47
Non-Hispanic other	1.32 (0.87-1.99)	.19
Education		
<High school	1 [Reference]	>.99
High school or equivalent	0.95 (0.69-1.31)	.77
>High school	1.42 (0.97-2.06)	.07
Not married vs married	1.43 (1.06-1.92)	.02
Nonmetropolitan area vs metropolitan	1.04 (0.77-1.40)	.79
Income poverty ratio Medicare threshold, % of FPL		
≤100	1 [Reference]	>.99
>100 to ≤120	0.71 (0.40-1.27)	.25
>120 to ≤135	0.54 (0.28-1.02)	.06
>135 to ≤200	1.12 (0.73-1.72)	.62
>200	0.68 (0.44-1.07)	.10
No supplemental insurance coverage vs supplemental coverage	1.13 (0.83-1.54)	.44
No. of chronic conditions		
0	1 [Reference]	>.99
1-2	1.15 (0.61-2.16)	.67
3-5	1.39 (0.77-2.49)	.28
≥6	1.86 (0.88-3.94)	.11
Functional limitations		
None	1 [Reference]	>.99
IADLs only	1.44 (0.97-2.12)	.07
ADLs		
1-2	2.18 (1.58-3.00)	<.001
3-4	2.94 (1.86-4.64)	<.001
5-6	3.27 (2.03-5.28)	<.001

Abbreviations: ADL, activities of daily living; FPL, Federal Poverty Level; IADL, instrumental activities of daily living; OR, odds ratio.

### Dissatisfaction With Specific Aspects of Care

Dissatisfaction with out-of-pocket costs paid for health care was the highest for all groups, ranging from 581 of 4250 respondents with no sensory impairment (weighted percentage, 14.7%) to 299 of 1557 respondents with vision impairment only (weighted percentage, 22.1%) (Figure). Those with dual sensory impairment had the highest percentage of dissatisfaction across all other outcomes; 191 of 2154 resepondents (weighted percentage, 9.6%) were dissatisfied with their doctors’ concern about their overall health. The highest percentage of dissatisfaction among those with hearing and vision impairment only, after out-of-pocket costs paid, was for information given about what was wrong (hearing, 143 of 2822 [weighted percentage, 5.3%]; vision, 113 of 1557 [weighted percentage, 7.4%]).

In fully adjusted models, those with dual sensory impairment had the highest odds of dissatisfaction compared with those without sensory impairment across outcomes (Table 3); the greatest odds of dissatisfaction among those with dual sensory impairment were for dissatisfaction with doctors’ concern with overall health (OR, 2.03; 95% CI, 1.55-2.66; *P* < .001). Those with hearing impairment only also had greater odds of dissatisfaction with their doctors’ concern (OR, 1.38; 95% CI, 1.03-1.86; *P* = .04). Having any kind of sensory impairment was associated with greater odds of dissatisfaction with the information given about what was wrong; compared with no sensory impairment, having dual sensory impairment (OR, 1.82; 95% CI, 1.40-2.37), hearing impairment (OR, 1.67; 95% CI, 1.29-2.17), or vision impairment (OR, 1.56; 95% CI, 1.18-2.08) were associated with dissatisfaction with the information provided about what was wrong. Those with vision impairment only and dual sensory impairment had higher odds of dissatisfaction with the ease to get to a doctor (vision only: OR, 1.63; 95% CI, 1.14-2.31; *P* = .008; dual sensory: OR, 1.69; 95% CI, 1.24-2.30; *P* = .002) and the out-of-pocket costs paid for health care (vision only: OR, 1.31; 95% CI, 1.07-1.61; *P* = .01; dual sensory: OR, 1.27; 95% CI, 1.04-1.54; *P* = .02).

**Table 3.** Dissatisfaction With Different Aspects of Care by Sensory Impairment Status in the 2017 Medicare Current Beneficiary Survey

	Unadjusted models		Adjusted models^a^	
	OR (95% CI)	*P* value	OR (95% CI)	*P* value
**Dissatisfaction with the information given about what was wrong**				
Impairment				
No sensory	1 [Reference]	>.99	1 [Reference]	>.99
Hearing only	1.69 (1.33-2.15)	<.001	1.67 (1.29-2.17)	<.001
Vision only	2.41 (1.80-3.23)	<.001	1.56 (1.18-2.08)	.003
Dual sensory	2.88 (2.25-3.69)	<.001	1.82 (1.40-2.37)	<.001
**Dissatisfaction with doctors’ concern with overall health rather than isolated symptoms/diseases**				
Impairment				
No sensory	1 [Reference]	>.99	1 [Reference]	>.99
Hearing only	1.34 (1.00-1.79)	.05	1.38 (1.03-1.86)	.04
Vision only	1.82 (1.34-2.49)	<.001	1.29 (0.93-1.80)	.13
Dual sensory	2.82 (2.21-3.59)	<.001	2.03 (1.55-2.66)	<.001
**Dissatisfaction with the ease to get to a doctor from home**				
Impairment				
No sensory	1 [Reference]	>.99	1 [Reference]	>.99
Hearing only	1.07 (0.74-1.56)	.71	1.01 (0.68-1.50)	.97
Vision only	2.52 (1.78-3.55)	<.001	1.63 (1.14-2.31)	.008
Dual sensory	2.84 (2.12-3.79)	<.001	1.69 (1.24-2.30)	.002
**Dissatisfaction with the out-of-pocket costs paid for health care**				
Impairment				
No sensory	1 [Reference]	>.99	1 [Reference]	>.99
Hearing only	1.01 (0.83-1.23)	.92	0.98 (0.80-1.20)	.86
Vision only	1.65 (1.35-2.01)	<.001	1.31 (1.07-1.61)	.01
Dual sensory	1.61 (1.33-1.95)	<.001	1.27 (1.04-1.54)	.02

Abbreviation: OR, odds ratio.

^a^ Models adjusted for age, sex, race/ethnicity, education, marital status, metropolitan area status, income poverty ratio Medicare threshold, supplemental health insurance, number of chronic conditions, and number of functional limitations.

After excluding Medicare beneficiaries younger than 65 years, the odds of dissatisfaction with doctors’ concern with overall health among those with hearing impairment and the odds of dissatisfaction with ease of getting to a doctor and out-of-pocket costs among those with vision impairment were attenuated and no longer statistically significant relative to no sensory impairment (eTable in the Supplement).

## Discussion

In a nationally representative sample of Medicare beneficiaries, those with dual sensory impairment had significantly higher odds of dissatisfaction with perceived quality of care relative to those without sensory impairment in a model adjusted for sociodemographic and health covariates. In secondary analyses, sensory impairment was associated with higher odds of dissatisfaction with aspects of communication with patients and access to care relative to those without sensory impairment, including information provided about what was wrong and ease to get to a doctor. To our knowledge, these findings represent the first analysis of dual sensory impairment and patient-reported satisfaction with care and may represent a modifiable pathway (ie, utilization of sensory aids) to improving care.

In the current study, dual sensory impairment was associated with greater dissatisfaction with quality of care. Those with hearing or vision impairment alone were approximately 30% more likely to report dissatisfaction, although this finding was not statistically significant. A small but growing body of work examining sensory loss and patient-reported satisfaction suggests hearing impairment is associated with reduced satisfaction with quality of care,^28,32^ and that vision or hearing impairment are associated with overall dissatisfaction with care.^30^ However, to our knowledge, no previous study focusing on patient-reported satisfaction with care has examined dual sensory impairment as an exposure and instead opted to explore hearing or vision alone. Although not directly comparable with our study, a study on family-reported perceptions of care among veterans receiving end-of-life care reported that family members of veterans with vision impairment were less likely to report excellent end-of-life care. However, when dual sensory impairment was taken into account, only families of veterans with dual sensory impairment were less likely to report excellent care.^29^ It is plausible that by omitting a category for those with dual sensory impairment in previous research, researchers have overlooked the subgroup driving associations between hearing or vision alone and satisfaction with care.

Similar to previous studies, dissatisfaction with care was a rare outcome, as less than 5% of the entire sample reported it.^32,33^ Respondents with activity limitations, unmarried respondents, and those in the younger age groups were more likely to be dissatisfied with care. In our sample, participants younger than 65 years were Medicare beneficiaries with a qualifying disability; this is consistent with previous studies^33,36^ that demonstrated that disability, and severity of disability, is associated with satisfaction with care. Having more functional limitations and severe disabilities may present additional barriers to care, while married individuals may potentially have a companion that can help them navigate the health care system and lessen the perceived barriers.

In secondary analyses, sensory impairment was associated with dissatisfaction with information provided as well as doctors’ concern with overall health rather than isolated symptoms and/or diseases. Notably, older adults with dual sensory impairment had the highest odds of dissatisfaction, followed by hearing impairment only. Previous studies^7,8,28,29,30,31^ have reported that both vision and hearing impairment are associated with poor communication with health care professionals and access to health information. This association may reflect the reliance on oral communication in health care interactions that are directly impacted by hearing impairment, while written materials that would be impacted by vision impairment, such as discharge instructions, are meant to augment the interaction. The loss of sensory substitution or compensation that comes with dual sensory impairment could affect communication at all levels. Problems communicating with health care professionals or following discharge information may potentially lead to higher readmission risk or longer hospitalizations, which have previously been associated with sensory impairment.

Medicare beneficiaries with vision impairment only and dual sensory impairment reported dissatisfaction with ease of getting to doctors from their homes. In sensitivity analyses limited to older adults, the association remained significant for the dual sensory impairment group only. Results specific to vision impairment are consistent with previous studies.^30,33^ To our knowledge, this is the first study demonstrating that those with dual sensory impairment had the greatest odds of dissatisfaction with ease of transportation to doctors among sensory groups relative to those without sensory impairment. People with vision impairment have identified physical access barriers and transportation needs as challenges to obtaining health care.^7,31^ In addition, it is possible the time demands of arranging transportation may create an extra barrier for adults with vision or dual sensory impairment, especially among those in the working-age group. Hearing impairment’s lesser limitation on independent transportation (ie, owning a license) may be reflected in the lack of association with ease of transportation in this study.

Those with vision impairment and dual sensory impairment, but not hearing impairment only, were also more likely than those without sensory impairment to report dissatisfaction with out-of-pockets costs paid. Dual sensory, vision, and hearing impairment have been individually linked with higher health care expenditures; however, little work has characterized satisfaction with out-of-pocket costs.^25,26^ Dissatisfaction with out-of-pocket costs paid among people with sensory impairment could reflect higher health care spending despite a poorer perceived health care experience because of communication and access barriers. Moreover, in a qualitative study, people with vision loss reported that transportation arrangements made because of physical access barriers can be costly^31^; the added cost of transportation may not be an issue for those with hearing impairment only, which could explain the lack of an association between hearing impairment and dissatisfaction with costs paid. Sensitivity analyses that excluded younger Medicare beneficiaries with a qualifying disability revealed that the association between vision impairment only and dissatisfaction with costs paid was no longer significant. In our sample, those with vision impairment only had the highest proportion of working-age adults; this may explain why the estimates for this group were affected the most. Moreover, working-age adults who qualify for Medicare may have other competing medical conditions or disabilities that may also be associated with out-of-pocket spending, thus resulting in more dissatisfaction.

Satisfaction with care is tied to the hospital value-based purchasing program under Medicare, in part through the Hospital Consumer Assessment of Healthcare Providers and Systems surveys.^37^ Sensory impairment may represent a target area to improve assessment survey scores. While not currently a general practice, screening patients for sensory impairment in different health care settings could help target those who may have specific communication or accessibility needs. In addition to providing or encouraging the use of sensory aids, potential compensatory interventions should target both hearing and vision impairment, as people with dual sensory impairment seem to be the ones driving the association between sensory impairment and dissatisfaction with care.

### Limitations

This study has limitations that should be considered when interpreting the results. First, respondents who did not interact with the health care system in the past year were excluded, and their behavior (including health care access in the past year) could be shaped by their satisfaction with care in previous years. Second, sensory impairment was based on self-report, and a broad definition was used to categorize it (ie, “a little trouble” or “a lot of trouble”). Moreover, only the use of hearing aids or glasses were accounted for as possible accommodation strategies. The outcomes were also subjective, and how a respondent reports sensory impairment may influence how they report satisfaction with care as well. While self-reported sensory impairment may be subject to bias, it is valuable in capturing people’s perception of their functional hearing and vision and consistent with a disability framework perspective.^38^ Moreover, people who reported difficulty seeing or hearing and perceived barriers to care and communication with health care professionals may potentially benefit from interventions that can improve communication or access, regardless of the severity of their impairment or clinical assessment. Last, we lacked information about region of residence, usual source of care, or health literacy, which may also affect the association between sensory impairment and satisfaction with care.

Future work should examine longitudinal associations, and more research is needed to detect specific communication and access barriers as well as other aspects of care that may be impacting the perception of overall quality of care among people with sensory impairment. Future studies could also assess if accompaniment to health care visits or presence of a caregiver modifies these associations. Analyzing Hospital Consumer Assessment of Healthcare Providers and Systems survey data by sensory impairment status, including dual sensory, could help examine if differences in satisfaction rates are enough to impact reimbursement, which may be important for hospital planning and spending.

## Conclusions

Sensory impairment, most notably dual sensory impairment, was associated with lower satisfaction with care in a nationally representative sample of Medicare beneficiaries. These findings have implications for the provision of patient-centered care for older adults with sensory impairment, and may impact health care planning and spending as Medicare ties reimbursement to patient satisfaction.

## Notes

Supplement. **eTable.** Multivariable-Adjusted Odds Ratios and 95% Confidence Intervals of Dissatisfaction with Different Aspects of Care by Sensory Impairment Status Among Medicare Beneficiaries 65 Years and Older, the Medicare Current Beneficiary Survey (2017)
