# Supplementary material for: Assessment of Sensory Impairment and Health Care Satisfaction Among Medicare Beneficiaries
Source: JAMA Netw Open. 2020 Nov 13;3(11):e2025522. doi: 10.1001/jamanetworkopen.2020.25522 (PMC7666423; doi:10.1001/jamanetworkopen.2020.25522)
Supplement: Supplement. — eTable. Multivariable-Adjusted Odds Ratios and 95% Confidence Intervals of Dissatisfaction with Different Aspects of Care by Sensory Impairment Status Among Medicare Beneficiaries 65 Years and Older, the Medicare Current Beneficiary Survey (2017) [file jamanetwopen-e2025522-s001.pdf]

Supplemental Online Content

Assi L, Shakarchi AF, Sheehan OC, Deal JA, Swenor BK, Reed NS. Assessment of sensory impairment and health care satisfaction among Medicare beneficiaries. *JAMA Netw Open*. 2020;3(11):e2025522. doi:10.1001/jamanetworkopen.2020.25522

eTable. Multivariable-Adjusted Odds Ratios and 95% Confidence Intervals of Dissatisfaction with Different Aspects of Care by Sensory Impairment Status Among Medicare Beneficiaries 65 Years and Older, the Medicare Current Beneficiary Survey (2017)

This supplemental material has been provided by the authors to give readers additional information about their work.

© 2020 Assi L et al. *JAMA Network Open.*

eTable. Multivariable-Adjusted Odds Ratios and 95% Confidence Intervals of Dissatisfaction with Different Aspects of Care by Sensory Impairment Status Among Medicare Beneficiaries 65 Years and Older, the Medicare Current Beneficiary Survey (2017)

	**All Medicare Beneficiaries (N=10,783)**		**Only Medicare Beneficiaries 65 Years and Older (N=8,944)**	
	AOR (95% CI)	*P value*	AOR (95% CI)	*P value*
**Dissatisfaction with the overall quality of medical care received**				
None	Reference		Reference	
HI only	1.31 (0.88, 1.95)	0.19	1.18 (0.79, 1.76)	0.43
VI only	1.17 (0.79, 1.74)	0.43	1.27 (0.76, 2.11)	0.37
DSI	1.52 (1.10, 2.10)	0.01	1.67 (1.14, 2.44)	0.01
**Dissatisfaction with the information given about what was wrong**				
None	Reference		Reference	
HI only	1.67 (1.29, 2.17)	<0.001	1.72 (1.27, 2.32)	<0.001
VI only	1.56 (1.18, 2.08)	0.003	1.81 (1.23, 2.66)	0.004
DSI	1.82 (1.40, 2.37)	<0.001	2.04 (1.47, 2.83)	<0.001
**Dissatisfaction with doctors' concern with overall health rather than isolated symptoms/diseases**				
None	Reference		Reference	
HI only	1.38 (1.03, 1.86)	0.04	1.34 (0.97, 1.87)	0.08
VI only	1.29 (0.93, 1.80)	0.13	1.29 (0.85, 1.98)	0.23
DSI	2.03 (1.55, 2.66)	<0.001	1.89 (1.40, 2.55)	<0.001
**Dissatisfaction with the ease to get to a doctor from home**				
None	Reference		Reference	
HI only	1.01 (0.68, 1.50)	0.97	0.96 (0.63, 1.47)	0.85
VI only	1.63 (1.14, 2.31)	0.008	1.19 (0.73, 1.94)	0.48
DSI	1.69 (1.24, 2.30)	0.002	1.71 (1.24, 2.36)	0.002
**Dissatisfaction with the out-of-pocket costs paid for health care paid**				
None	Reference		Reference	
HI only	0.98 (0.80, 1.20)	0.86	0.92 (0.73, 1.16)	0.49
VI only	1.31 (1.07, 1.61)	0.01	1.26 (0.96, 1.64)	0.10
DSI	1.27 (1.04, 1.54)	0.02	1.30 (1.04, 1.62)	0.02

Abbreviations: AOR, adjusted odds ratio; CI, confidence interval; HI, hearing impairment; VI, vision impairment; DSI, dual sensory impairment.

All models adjusted for age, sex, race/ethnicity, education, marital status, metropolitan area status, income poverty ratio Medicare threshold, supplemental health insurance, number of chronic conditions, number of functional limitations.

© 2020 Assi L et al. *JAMA Network Open.*
